# Interactions between Enteric Bacteria and Eukaryotic Viruses Impact the Outcome of Infection

**DOI:** 10.3390/v10010019

**Published:** 2018-01-03

**Authors:** Angela K. Berger, Bernardo A. Mainou

**Affiliations:** 1 Department of Pediatrics, Emory University School of Medicine, Atlanta, GA 30322, USA; akberge@emory.edu; 2 Children’s Healthcare of Atlanta, Atlanta, GA 30322, USA

**Keywords:** virus, bacteria, intestines, microbiota, poliovirus, norovirus, mouse mammary tumor virus, reovirus, virus and host interactions

## Abstract

Enteric viruses encounter a multitude of environments as they traverse the gastrointestinal tract. The interaction of enteric eukaryotic viruses with members of the host microbiota impacts the outcome of infection. Infection with several enteric viruses is impaired in the absence of the gut microbiota, specifically bacteria. The effects of bacteria on virus biology are diverse. Poliovirus capsid stability and receptor engagement are positively impacted by bacteria and bacterial lipopolysaccharides. Norovirus utilizes histo-blood group antigens produced by enteric bacteria to attach and productively infect B cells. Lipopolysaccharides on the envelope of mouse mammary tumor virus promote a tolerogenic environment that allows for the establishment of viral persistence. Reovirus binds Gram negative and Gram-positive bacteria through bacterial envelope components to enhance virion thermostability. Through the direct engagement of bacteria and bacterial components, viruses evolved diverse ways to impact the outcome of infection.

## 1. Introduction

**Figure 1:**
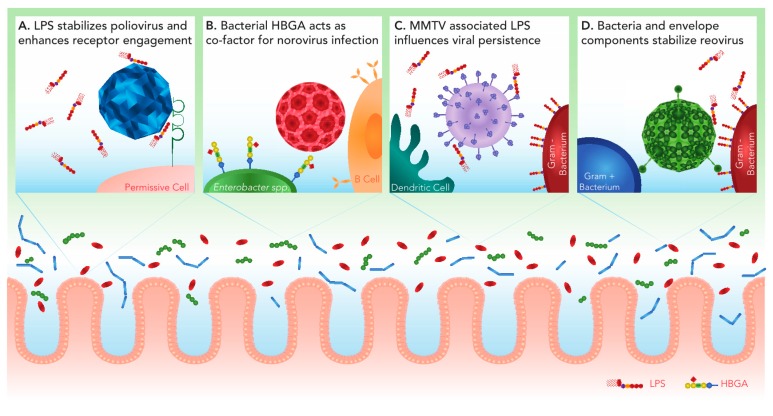
Viral interactions with intestinal bacteria positively impact infection. Within the intestinal lumen of the intestine, enteric viruses encounter a diverse microbiota and must traverse the mucus layer to reach the host epithelium. Some enteric viruses have evolved to interact with the gut microbiota to facilitate infection. (**A**) Polysaccharides including bacterial lipopolysaccharide (LPS) increase the thermostability of poliovirus. In addition to stability, LPS enhances poliovirus engagement of the cellular poliovirus receptor (PVR/CD155), leading to increased infectivity. (**B**) Histo-blood group antigen (HBGA)-expressing bacteria, including *Enterobacter* spp., facilitate human norovirus attachment and infection of B cells. (**C**) MMTV-bound LPS stimulates TLR4 on dendritic cells and macrophages. This leads to downstream signaling that results in the production of immunosuppressive cytokine IL-10, which allows the establishment of persistent viral infection. (**D**) Reovirus can associate with both Gram positive and Gram negative bacteria. The presence of the major bacterial envelope components LPS or peptidoglycan (PG) enhance viral thermostability resulting in increased attachment and infection of target cells.

The context in which viruses interact with their hosts is a key determinant of the outcome of viral infection. Innate and adaptive immune responses are important in the control of virus infection and eventual clearance of the virus from the host. The host microbiota can also impact the outcome of viral infection. Enteric viruses appear to be especially adept at directly and indirectly benefitting from the presence of microbes in the gastrointestinal tract. The evolution of enteric viruses to utilize the gut microbiota during infection is not surprising. The gastrointestinal tract is populated by a complex environment that includes a rich microbial milieu [1,2]. During oral infection, enteric viruses must navigate diverse surroundings including changing cellular landscapes, altered pH and oxygen levels and diverse microbial communities [1,2]. The small and large intestines play essential roles in enteric virus infection. In the small intestine viruses encounter changing pH levels (pH of 6.6 in the duodenum, pH of 7.3 in the terminal ileum), high oxygen levels (oxygen tension of 32 torr in the duodenum), discontinuous mucus levels where the villus tips are not always covered by mucus, low bacterial loads (10^2^ cfu g^−1^) and relatively low bacterial diversity [3,4,5]. In the large intestine, viruses encounter pH ranges of 6.4–7.0 in the colon [3], low oxygen levels (P_O2_ values less than 1 mm Hg and oxygen tension of 11 torr in the colon), two mucus layers (an outer loose layer and a firm inner mucus layer that is attached to the epithelium), large bacterial loads (10^11^ cfu g^−1^) and greater bacterial diversity [1,2,4,5,6]. The changing microenvironments of the small and large intestine affect the microbial communities within these organs. Fast growing facultative anaerobes like certain *Clostridium* spp. and members of the Proteobacteria that can tolerate bile acids and antimicrobial peptides predominate in the small intestine [7]. In the cecum and colon, lower levels of antimicrobials, low oxygen levels, and availability of complex polysaccharides that were not broken down in the small intestine promote the growth of fermentative polysaccharide-degrading anaerobes like the Bacteroidaceae and Clostridia [8,9]. Viruses use different aspects of the microbial communities they encounter in the gut to survive these fluctuating environments. Poliovirus and mammalian orthoreovirus (reovirus) utilize bacteria and bacterial envelope components to enhance virion stability [10,11,12]. Poliovirus and norovirus use components of the microbiota to affect attachment and infectivity [12,13]. Mouse mammary tumor virus (MMTV) uses lipopolysaccharide (LPS) bound to its envelope to promote a microenvironment that is more conducive for the establishment of viral persistence [14]. Enteric microbiota can also greatly impact the innate and adaptive immune responses to infection, including the production of antibodies and interferon (IFN) λ [15,16]. It is unclear to what extent enteric virus infection impacts microbial communities in the gut and if interactions between eukaryotic viruses and enteric bacteria are also beneficial to the bacteria. Here, we focus on how the direct interactions between enteric bacteria and viruses impact the outcome of viral infection (Figure 1).

## 2. Poliovirus

Poliovirus is a non-enveloped, single stranded RNA virus in the *Enterovirus* genus of the *Picornaviridae* family of viruses. Poliovirus is the causative agent of poliomyelitis, a paralytic disease that was first described in the 1800s [17]. Poliovirus is transmitted via fecal-oral routes, establishes infection, and can spread to the central nervous system (CNS) by hematogenous and neural routes [17,18]. Poliovirus cell entry is mediated by the cellular poliovirus receptor (PVR or CD155) [19]. PVR is expressed in many cells and tissues, including the intestinal epithelium, M cells, and germinal centers in Peyer’s patches [20,21]. Primary infection occurs primarily in the oropharyngeal and intestinal mucosa. Mice overexpressing PVR in the intestinal epithelium are not susceptible to oral poliovirus infection, suggesting factors beyond receptor expression determine in vivo poliovirus tropism [20,22].

As a pathogen that is transmitted through fecal-oral routes, polioviruses must traverse the microbe-rich environment of the gastrointestinal tract. In transgenic mice expressing PVR, depletion of the enteric microbiota with antibiotics before oral inoculation with poliovirus decreases mortality compared to control animals [11]. In addition, the reintroduction of fecal bacteria into mice with a depleted microbiota results in similar mortality compared with untreated mice. Antibiotic-treated mice also shed significantly less poliovirus than untreated animals. Interestingly, treatment of mice with antibiotics at the time of inoculation does not affect poliovirus pathogenesis, indicating that the effects observed in antibiotic-treated mice are not due to direct antiviral effects of the antibiotics. As such, the presence of bacteria in the gut is essential for efficient poliovirus-induced disease. While the presence of enteric bacteria is essential for the first round of infection in the gut, depletion of enteric bacteria after the establishment of infection does not significantly impact poliovirus disease.

The mechanism that underlies bacterial enhancement of poliovirus pathogenesis is multifactorial. Incubation of poliovirus at 37 °C or 42 °C with the feces of antibiotic-treated or germ-free mice decreases virus infectivity compared with virus that was incubated with the feces of untreated mice or with Gram negative or Gram-positive bacteria, LPS, or peptidoglycan (PG) [11,12]. These data suggest that poliovirus infectivity is positively affected by the presence of bacteria. Poliovirus infectivity also can be positively impacted by *N*-acetylglucosamine (GlcNAc)-containing polysaccharides, like mucin or chitin. Incubation of poliovirus at higher temperatures results in conformational changes that result in the premature release of the viral RNA from the particle, with RNA release and capsid denaturation peaking at 46 °C [12,23]. Incubation of poliovirus with LPS increases capsid thermostability by 3 °C, shifting RNA release and capsid denaturation to 49 °C [12]. Beyond enhancing capsid thermostability, incubation of poliovirus with LPS enhances binding of the virus to PVR, which results in enhanced attachment to host cells [12]. Addition of LPS or PG also enhances the stability of other *Picornaviridae*, including Coxsackievirus A21 and B5 and echovirus 30 [24].

In comparison to the Mahoney strain of poliovirus, which has enhanced thermostability in the presence of LPS, the thermostability of Sabin serotype 1 poliovirus, the strain used in the oral poliovirus vaccine, is not altered by LPS [12]. The Mahoney strain differs from the Sabin strain by 12 amino acids in capsid proteins [25]. The amino acid at position 99 on the VP1 capsid protein plays an important role in the ability of the virus to bind LPS. Introduction of a lysine residue at this position, which is present in the Sabin strain, into Mahoney poliovirus (T99K) reduces LPS binding and impairs LPS-mediated enhancement of virion stability but surprisingly has no effect on attachment to cells compared to wild-type Mahoney poliovirus [12]. In vivo, wild-type and T99K polioviruses replicate to similar levels and induce morbidity to similar levels. However, T99K poliovirus has decreased stability in animal feces compared to wild-type, suggesting that LPS-mediated stability plays an important role in fecal-oral transmission between hosts. Studies with poliovirus show how the host microbiota can impact various aspects of virus infection, including capsid stability, receptor engagement, replication, shedding and pathogenesis.

## 3. Norovirus

Norovirus (NoV) is a non-enveloped, positive sense, single stranded RNA viruses in the *Calicivirus* family. Human NoVs (HuNoVs) are the leading cause of gastroenteritis, causing 19–21 million symptomatic infections in the United States and are the leading cause of severe childhood diarrhea [26]. The HuNoV major capsid protein, VP1, can self-assemble into virus like particles (VLPs) that are similar to native virions and is the major target of the host adaptive immune response [27,28]. HuNoV infects the intestinal epithelium, dendritic cells, macrophages and T cells of immunocompromised people and animal models [29,30,31] as well as B cells [13,32] and human intestinal enteroids [33]. A model for the study of HuNoV, Murine NoV (MuNoV), crosses the intestinal epithelial barrier by traversing through microfold (M) cells in Peyer’s patches [34,35] and can efficiently infect macrophages, dendritic cells, B cells, and T cells [13,28,32,36]. MuNoV utilizes the proteinaceous cellular proteins CD300ld and CD300lf to infect cells, although it is unclear if HuNoV can use these receptors or the human homologues to infect cells [37,38]. Intestinal epithelial cells serve as the reservoir for MuNoV shedding and persistence [39]. Intriguingly, it is the nonstructural viral protein NS1, likely through its antagonism of IFN-λ, that determines tropism for intestinal epithelial cells.

HuNoVs and MuNoVs utilize a variety of glycans to infect cells, although the mechanism by which glycans mediate infection is not completely clear [40,41,42,43]. The utilization of different glycans by MuNoV strains can alter cell and tissue tropism. The ability of the CR3 strain to infect cells in the large intestine are linked to a glycan-binding site in the VP-1 capsid protein [42]. HuNoVs utilize different histo-blood group antigens (HBGA) to attach to cells [41,43,44,45,46,47,48]. *Enterobacter* spp. bacteria, including *E. cloacae*, express HBGA-like substances and HuNoVs associate with HBGA-expressing *E. cloacae* [49]. HuNoVs can also associate with several bacterial species found in human stool [50], although it is not known if HBGA-like substances are necessary for interactions with all bacterial species. MuNoV and HuNoV can productively infect B cells [13,32]. HuNoV infection of B cells requires the supplementation of H-type HBGA or incubation with *E. cloacae* [13]. In contrast to poliovirus, LPS or H-type HBGA-negative *E. coli* do not promote HuNoV infection of B cells. Sapovirus, a member of the *Caliciviridae* family, does not utilize HBGA antigens to attach to cells. Instead, sapoviruses use O-linked sialic acids [51]. Through the use of a variety of polysaccharides, caliciviruses have evolved a conserved mechanism to attach to host cells and, in part, determine their cellular and tissue tropism.

Similar to that observed with poliovirus, depletion of enteric bacteria with antibiotics prior to infection decreases MuNoV infectivity in the intestines [13,15]. Depletion of the enteric microbiota affects infection in the intestines but does not affect replication in secondary sites of infection [15]. These data suggest that glycans produced by enteric bacteria are necessary for efficient NoV infection of cells in the intestines but not for dissemination to secondary sites. At least in humans, NoV susceptibility is dependent on a mixture of host genetics, especially those involving fucosyltransferase 2 (FUT2) [52] and the composition of the gut microbiota [53]. FUT2 transfers fucose to HBGA precursors and individuals with nonfunctional FUT2 are HBGA secretor-negative [52]. In humans, the presence of *Faecalibacterium* and *Ruminococcus* spp. in the gut negatively correlate with HuNoV infectivity [53]. Infection of intestinal epithelial cell organoids does not require bacteria for infection but the secretor status is still important for infection with some HuNoV strains [33]. Moreover, infection of intestinal epithelial cell organoids with some HuNoV strains requires pretreatment of cells with bile. Interestingly, sapovirus infection of porcine kidney cells is also enhanced by bile [54,55], suggesting that the *Caliciviridae* have evolved mechanisms to utilize bile in the intestinal tract to infect cells.

Bile has bactericidal properties against some strains of bacteria. Animals fed excess bile have increased levels Firmicutes and decreased levels of Bacteroidetes [56]. As such, it is possible that that NoVs utilize cofactors from bile-resistant bacteria to promote infection in the gut. In mice, establishment of MuNoV persistence is impaired by the administration of a cocktail of ampicillin and vancomycin but not individual antibiotics [15]. The presence of bacteria in the gut suppresses the Type III IFN response, which is involved in controlling the establishment of persistent infection by MuNoV [15,57]. As such, bacteria in the gut can affect the outcome of norovirus infection by direct and indirect means. It remains to be determined if HuNoVs use a CD300-like proteinaceous receptor to infect cells. MuNoVs that utilize CD300lf do not require secretor HBGAs to infect cells [37], suggesting that different mechanisms could be at play in how the host microbiota regulate NoV infection.

## 4. Mouse Mammary Tumor Virus

Mouse mammary tumor virus (MMTV) is a murine betaretrovirus that causes mammary epithelial cell tumors [58]. MMTV is transmitted to nursing pups through the ingestion of maternal milk containing the virus [59]. Commonly used inbred mice contain exogenous and endogenous copies of MMTV in their genome, although most copies of endogenous MMTV do not produce infectious virus [58]. Once ingested, MMTV infects dendritic cells in the gut and spreads to B and T cells in Peyer’s patches. MMTV disseminates to B and T cells in other lymphoid organs, where virus amplification occurs. These cells also serve as a reservoir for the virus during persistent infection [58]. MMTV-infected lymphocytes can migrate to the mammary gland where MMTV infects mammary epithelial cells [58,60]. Although MMTV oncogenesis occurs at a distant site from the gut, the gastrointestinal tract plays a key role in MMTV infection, the establishment of persistence, and transmission.

Establishment of persistent MMTV infection from virus transmitted through maternal milk requires Toll-lie receptor 4 (TLR4), the LPS pattern recognition receptor, and downstream production of interleukin-10 (IL-10) [61]. TLR4 is activated during MMTV infection of dendritic cells and macrophages [61]. MMTV found in maternal milk associates with LPS, likely through the incorporation of LPS-binding proteins CD14, MD2 and TLR4 on the viral envelope [14,62]. TLR4 signaling in dendritic cells and macrophages stimulates the production of IL-6, which then induces IL-10 production by B cells [14]. Not surprisingly, the association of MMTV with LPS is necessary for virus-induced IL-10 secretion in splenocytes and LPS-associated with the virion induces more robust IL-6 production than virus-free LPS [62]. IL-10 in turn inhibits macrophage activation and promotes B cell growth. In this immunosuppressive environment, MMTV can establish viral persistence. As such, MMTV utilizes components of the host microbiota to regulate the host response to viral infection to its advantage, effectively utilizing bacterial LPS as a protective cloak to evade host immune defenses.

## 5. Reovirus

Reovirus is a non-enveloped, segmented dsRNA virus in the *Reoviridae* family [63]. The *Reoviridae* also include rotavirus [64], a leading causative agent of diarrhea in infants and blue tongue virus, the causative agent of bluetongue disease in ruminants [65]. Most humans are infected with reovirus via respiratory or fecal-oral routes during childhood, although infection seldom results in disease [63,66,67,68,69]. In some cases, reovirus enteric infection can induce loss of tolerance to dietary antigens by affecting intestinal immune homeostasis [70]. Within the murine intestinal tract, reovirus is taken up and transported by M cells to underlying Peyer’s patches and infects intestinal epithelial cells [34,71,72,73,74]. Virus then disseminates from the gut to secondary sites of infection via hematogenous and neural routes [72,73,75,76]. Subsequent infection of intestinal epithelial cells plays an important role in virus shedding [34,77].

Similar to that observed with poliovirus and norovirus, depletion of the enteric microbiota with antibiotics prior to infection negatively impacts reovirus infection of the intestines [11]. In the absence of enteric bacteria, reovirus-induced intestinal pathology is also decreased [11]. Rotavirus infection and virus-induced pathology is similarly impaired in mice with a depleted enteric microbiota [16], indicating an important role for enteric bacteria in the infection and pathogenesis of *Reoviridae* viruses. In vitro, reovirus associates with Gram positive and Gram negative bacteria in a serotype-independent manner [10]. The association of reovirus with bacteria enhances virion thermostability. This effect is likely mediated through interactions with bacterial envelope components as LPS and PG, a major component of the cell envelope of Gram positive bacteria [78], also enhance reovirus virion thermostability [10]. LPS and PG enhance virion thermostability of both virions and infectious subvirion particles (ISVPs). ISVPs are produced during oral infection when the virus encounters intestinal proteases and contribute to the initial round of infection in the intestines [79]. Interestingly, although PG is as efficient as Gram positive bacteria at enhancing reovirus thermostability, LPS is not as efficient at enhancing reovirus thermostability as Gram negative bacteria. These data suggest that other factors beyond LPS in Gram negative bacteria influence virion thermostability.

How bacteria and the bacterial envelope enhance reovirus thermostability is unclear. When reovirus is incubated at temperatures ranging from 23 °C to 37 °C in the absence of bacteria or bacterial envelope components, virions are impaired in their ability to attach to cells [10]. The impairment in attachment is alleviated when virions are incubated with bacteria or bacterial envelope components. Interestingly, lipoteichoic acid, a component of the Gram positive bacterial envelope [78], also enhances the thermostability of reovirus strain Type 3 Dearing (T3D) but not reovirus strain Type 1 Lang (T1L). This suggests that there are strain-specific differences in how and what kinds of bacterial components are engaged to enhance the biophysical properties of the virions. It remains unclear how these differences affect the pathogenesis of each strain in vivo. The effects of bacteria and bacterial envelope components do not appear to significantly affect other aspects of the reovirus replication cycle. This includes cell entry kinetics and the efficiency with which reovirus-specific antibodies impair infection [10]. Together, these data suggest that bacteria impact the biophysical properties of reovirus virions and ISVPs, which are likely to affect the efficiency of enteric infection. IFN λ plays an important role in reovirus infection in the gut and enteric bacteria suppress the basal levels of expression of this cytokine [57]. As such, enteric bacteria are likely to affect reovirus infection in the gut through direct interactions with the virions and by modulating the host response to infection.

## 6. Conclusions

It is not surprising that hosts and pathogens have evolved to use the enteric microbiota to their benefit. Enteric viruses navigate through environments rich in microbes. Here, we discuss how poliovirus, norovirus, mouse mammary tumor virus, and reovirus use bacteria and bacterial envelope components to enhance various aspects of their replication, from cell attachment, to infection, and establishment of persistence. While bacteria impact similar aspects of virus biology (e.g., enhanced stability), they can also have multifactorial effects on viruses. Although we focus here on how enteric microbes directly impact viral infection, the host microbiota also affects the outcome of infection through its modulation of host immune responses to infection [15,16,57,80]. It is not well understood how specific microbiota at distinct sites within the gut impact viral infection. It is also not clear how other members of the intestinal microbiota, like fungi, alter the outcome of viral infection. Intriguingly, the presence of *Talaromyces* fungus in the midgut of mosquitoes renders them more permissive to infection by dengue virus [81]. Obtaining a better understating of the interactions between hosts, host microbes, and pathogens is likely to identify new aspects of host and virus biology and inform the development of novel therapeutics.
